# Effect of COVID-19 on Anti-S Antibody Response in Healthcare Workers Six Months Post-Vaccination

**DOI:** 10.3390/vaccines9111325

**Published:** 2021-11-15

**Authors:** Robert Flisiak, Małgorzata Pawłowska, Magdalena Rogalska-Płońska, Monika Bociąga-Jasik, Krzysztof Kłos, Anna Piekarska, Dorota Zarębska-Michaluk

**Affiliations:** 1 Department of Infectious Diseases and Hepatology, Medical University of Białystok, 15-540 Bałystok, Poland; pmagdar@gmail.com; 2 Department of Infectious Diseases and Hepatology, Faculty of Medicine, Collegium Medicum in Bydgoszcz, Nicolaus Copernicus University, 85-067 Bydgoszcz, Poland; mpawlowska@cm.umk.pl; 3 Department of Infectious and Tropical Diseases, Medical College, Jagiellonian University, 31-007 Krakow, Poland; monika.bociagajasik@gmail.com; 4 Department of Infectious Diseases and Allergology, Military Institute of Medicine, 04-141 Warsaw, Poland; kklos@wim.mil.pl; 5 Department of Infectious Diseases and Hepatology, Medical University of Łódź, 90-419 Łódź, Poland; annapiekar@gmail.com; 6 Department of Infectious Diseases, Jan Kochanowski University, 25-317 Kielce, Poland; dorota1010@tlen.pl

**Keywords:** COVID-19, SARS-CoV-2, vaccination

## Abstract

The current study aimed to determine to what extent prior COVID-19 infection affects the response of specific antibodies following vaccination. The study involved 173 healthcare professionals who completed the two-dose vaccination course with BNT162b2, including 40 who previously experienced clinical COVID-19. The levels of anti-SARS-CoV-2 S1S2 IgG (anti-S) and, in some cases, anti-SARS-CoV-S-RBD IgG (anti-S-RBD) were determined six months after complete vaccination. A level exceeding the cut-off values for both anti-S and anti-S-RBD was observed in 100% of subjects, but after setting the analysis to 5- and 10-fold cut-off levels, the percentage of subjects meeting this criterion was significantly higher for anti-S-RBD. The 100-fold cut-off level was achieved by only 21% and 16% for anti-S and anti-S-RBD, respectively. Anti-S and anti-S-RBD levels above ten times the positive cut-off were respectively observed in 91% and 100% individuals with a history of COVID-19, while among those without COVID-19, these values were 64% and 90%, respectively. Significantly higher incidence of values above 10 and 100 times the cut-off became apparent among people with a history of COVID-19. In conclusion, vaccination against COVID-19 following infection with the disease provides higher levels of specific antibodies 6 months after vaccination than those of individuals without a history of the disease, which supports the use of a booster dose, particularly for those who have not experienced SARS-CoV-2 infection.

## 1. Introduction

COVID-19 vaccines, which became available at the very end of 2020, have brought new hope to the fight against the pandemic [1]. The vaccines have been shown to have a good safety profile and efficacy against symptomatic SARS-CoV-2 infection [2,3,4,5]. The production of specific antibodies starts a few weeks after the first dose, but a second dose is required for optimal protection against COVID-19 [6,7,8]. As recently shown among those hospitalized due to COVID-19 in the first half of 2021, infections among vaccinated people are sporadic [9]. Recent studies suggest that humoral immunity against SARS-CoV-2, associated with natural infection or vaccination, may persist for more than six months, but specific antibody levels decline over time [7]. However, we must bear in mind that a large proportion of those vaccinated had previously experienced COVID-19 even before vaccination, gaining an additional immunogenic stimulus that could imitate the third dose of the vaccine. Unfortunately, the level of antibodies providing an effective barrier against SARS-CoV-2 infection has not yet been established. Despite the attempts to standardize the quantification of the level of antibodies, the tests of individual manufacturers present their concentration in various reference ranges and units. This makes it difficult to compare test results and conduct retrospective multicenter, real-world analyses, which do not use a central laboratory.

The current study aimed to determine to what extent prior COVID-19 infection affects the response of specific antibodies following vaccination.

## 2. Materials and Methods

The retrospective study involved 173 health workers (30 men and 143 women) aged 50.1 ± 10.3 years from six hospitals who completed an entire two-dose vaccination course (21 days apart) with BNT162b2 (BioNTech, Mainz, Germany and Pfizer, New York, USA) between January 18 and March 18, 2021. Of these, 40 had previously experienced symptomatic COVID-19 (C+) (mean: 102 ± 90 days), which was not the case for the remaining 133 participants (C−). The level of anti-spike (anti-SARS-CoV-2 S1S2 IgG; anti-S) antibodies was determined six months (+/−1 month) after the second vaccination dose. In addition, in some cases, anti-spike receptor binding domain antibodies (anti-SARS-CoV-S-RBD IgG; anti-S-RBD) were additionally measured. The study included consecutive anonymous samples obtained from volunteer hospital workers available six months (+/−1 month) after the end of the vaccination course. Due to the retrospective and anonymous nature of the study, it did not require authorization. The antibody level was measured using different methods and different normal ranges. The antibody levels are shown as multiple cut-off values determined for a positive result for a given diagnostic method. This was due to the fact that the tests were carried out in different hospitals whose laboratories used reagents from various manufacturers. Unfortunately, to date, the producers have not standardized the units and the way of interpreting the results. However, for each test, the manufacturers provide a cut-off value above which the result is considered positive. In an attempt to ensure comparability of the results obtained using different methods, we considered this cut-off value as a kind of universal unit and the multiple of its excess as the level of antibodies observed in a given person. Statistical analysis was performed using the chi-square test.

## 3. Results

**Figure 1:**
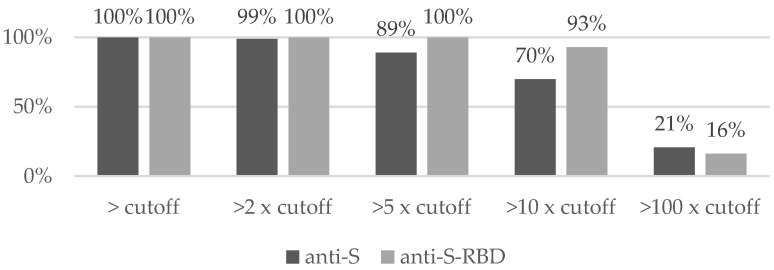
The proportion of vaccinated participants with anti-S and anti-S-RBD antibody levels greater than multiples of the cut-off determined the positive result.

A level exceeding the cut-off values for both anti-S and anti-S-RBD antibodies was observed in 100% of subjects, and almost the same result was obtained when doubling the cut-off level (Figure 1). However, after setting the analysis to 5- and 10-fold cut-off levels, the percentage of subjects meeting this criterion was significantly higher for the anti-S-RBD measurement and exceeded 90%. This difference disappeared at the 100-fold cut-off level, with anti-S and anti-S-RBD achieving only 21% and 16%, respectively (Figure 1).

**Figure 2:**
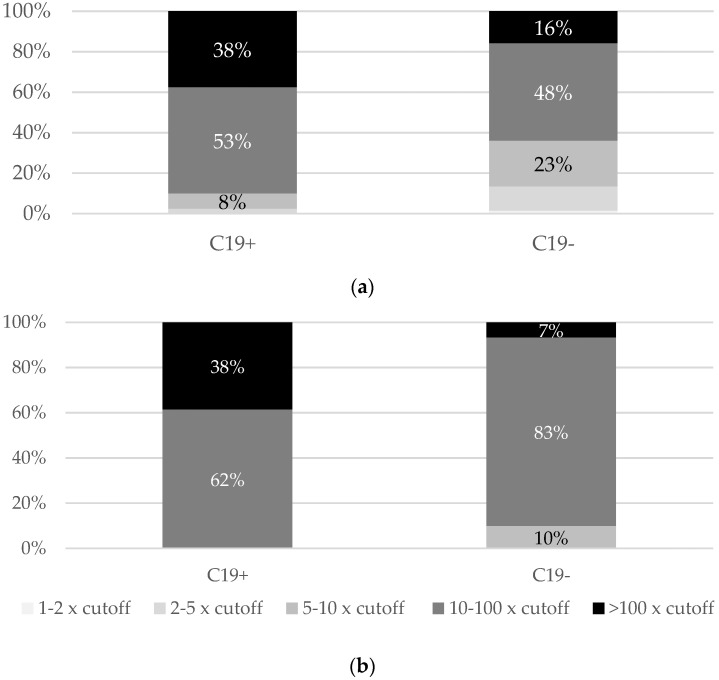
Prevalence of anti-S (**a**) and anti-S-RBD (**b**) antibodies within the ranges of the cut-off the positive result multiples in individuals with (C19+) and without (C19−) history of clinical COVID-19.

Most people with a history of COVID-19 had levels of both anti-S (91%) and anti-S-RBD (100%) above ten times the positive cut-off, while among those without a history of COVID-19, the percentages of such the values were lower (64% and 90%, respectively) (Figure 2).

A comparison of data between subjects with a clinical history of COVID-19 and those without it did not show a statistical difference in the frequency of anti-S values above the cut-off and double the positive cut-off (Table 1). However, as shown in Table 1, a significantly higher incidence among people with a history of COVID-19 became apparent for anti-S values set above 5, 10, and 100 times the cut-off value. A similar tendency was demonstrated for anti-S-RBD but only for 10 and 100 times the cut-off value (Table 2).

## 4. Discussion

In a placebo-controlled study evaluating the long-term efficacy of the BNT162b2 vaccine, carried out after the publication of the registration study results, the effectiveness of the vaccine against COVID-19 at six months of follow-up was 91%, while that against severe disease was 97% [2,3]. In turn, in studies carried out in clinical practice, vaccination protected 20%–47% of the subjects against infection within six months and 90%–96% of the subjects against a severe course of the disease requiring hospitalization and posing a threat to life [6,8]. On the other hand, we know that neutralizing antibodies persisted in 89% of the subjects and anti-spike antibodies in 97% of the subjects for at least 13 months after infection, but this figure decreased to 36% for anti-nucleoprotein antibodies [10]. These studies also showed that there are more neutralizing antibodies after a severe course of the disease [10].

According to data provided by Dimeglio et al. [11], sufficient protection of 89% can be obtained with a neutralizing antibody level of 141 BAU/mL, which is approximately ten times the level considered in this study as the limit for positive results. In our study, only 64% of vaccinated people with no history of COVID-19 exceeded this anti-S antibody level, in contrast to 90% of those with a history of the disease. Therefore, it can be considered that COVID-19 enhances the humoral response and can be equivalent to a booster dose of the vaccine. This hypothesis is supported by the six-month observations in the registration study of BNT162b2. The clinically evident COVID-19 was less common among the placebo recipients with positive N-binding antibodies at trial (1.3%) than placebo recipients among those without infection at trial entry (4.7%). Based on these data, it can be concluded that the protective effect of the previous symptomatic infection is approximately 73% [3].

Long-term follow-up would be needed to confirm the possible thesis that a booster dose of the vaccine is not required in previously infected individuals. However, we must bear in mind that we do not know the actual protective level of antibodies, so even higher concentrations of antibodies in this group of people do not guarantee safety. As the health of healthcare professionals is particularly important, until the results of long-term observation are obtained, the booster dose should be recommended in this group, regardless of the condition of previous infection, even if we assume that it may be equivalent to a booster dose.

The limitations of the current study are the relatively small number of samples, the inability to compare the absolute values of antibody concentrations due to the use of different methods of determination, and the lack of standardization. It should also be borne in mind that the study covers only specific humoral immunity, which may be significantly influenced by cellular immunity and the associated immune memory. Another limitation is the sole reliance on interviews when assessing the history of COVID-19. Further observation of the behavior of antibody levels is advisable to analyze the possibility of their disappearance. However, this may not be feasible due to the highly likely uptake of a booster dose by study participants.

## 5. Conclusions

Vaccination against COVID-19 in people who have previously experienced the disease provides higher levels of specific antibodies, especially against the spike receptor binding domain, six months after vaccination than those in those who have not experienced clinical COVID-19. The lower level of antibodies in people who have not had SARS-CoV-2 infection supports the use of a booster dose imitating the COVID-19 disease.

## Figures and Tables

**Table 1:** Prevalence of anti-S antibodies at the levels exceeding multiples of the cut-off determined for the positive result in individuals with and without a history of clinical COVID-19; NS—not significant.

Multiples of the Cut-Off	History of COVID-19 *n* = 40	No History of COVID-19 *n* = 133	*p*
>cutoff	40 (100%)	133 (100%)	NS
>2 × cutoff	40 (100%)	131 (98%)	NS
>5 × cutoff	39 (98%)	115 (86%)	0.0005
>10 × cutoff	36 (90%)	85 (64%)	<0.0001
>100 × cutoff	15 (38%)	21 (16%)	<0.0001

**Table 2:** Prevalence of anti-S-RBD antibodies at the levels exceeding multiples of the cut-off determined for the positive result in individuals with and without a history of clinical COVID-19; NS—not significant.

Multiples of the Cut-Off	History of COVID-19	No History of COVID-19	*p*
	*n* = 13	*n* = 30	
>cutoff	13 (100%)	30 (100%)	NS
>2 × cutoff	13 (100%)	30 (100%)	NS
>5 × cutoff	13 (100%)	30 (100%)	NS
>10 × cutoff	13 (100%)	27 (90%)	0.0008
>100 × cutoff	5 (38%)	2 (7%)	<0.0001

## Data Availability

The data presented in this study are available from the corresponding author on reasonable request.
